# Comparative Study on Milk Production, Milk Components, Feed Intake and Efficiency Parameters of Fleckvieh (German Simmental), Brown Swiss and Fleckvieh × Red Holstein Dairy Cows

**DOI:** 10.1111/jpn.14124

**Published:** 2025-05-19

**Authors:** Annika Bosch, Armin Manfred Scholz, Franziska Blümel, Johann Ertl, Hubert Spiekers, Thomas Ettle

**Affiliations:** ^1^ Bavarian State Research Center for Agriculture (LfL) Institute for Animal Nutrition and Feed Management Poing‐Grub Bavaria Germany; ^2^ Livestock Center Oberschleißheim, Veterinary Faculty University of Munich (LMU) Oberschleißheim Bavaria Germany; ^3^ Bayerische Staatsgüter (BaySG) Bavarian State Research Center for Agriculture in Achselschwang Utting am Ammersee Bavaria Germany; ^4^ Arbeitsgemeinschaft Süddeutscher Rinderzucht‐ und Besamungsorganisationen e.V. (ASR) Poing‐Grub Bavaria Germany

**Keywords:** Braunvieh, breed, dairy, feed efficiency, milk urea

## Abstract

The aim of this study was to compare milk production, milk components and feed intake of Fleckvieh, Brown Swiss and Fleckvieh × Red Holstein dairy cows kept under identical feeding and management conditions. The study examined efficiency parameters in relation to feed, energy, protein, and metabolic weight of the three breeds. Additionally, the changes of body condition throughout the lactation were assessed using body condition score and back fat thickness. Data collected from 24 feeding trials conducted at the Bavarian State Research Center for Agriculture between 2011 and 2023 were compiled. Totally, 99,288 daily observations from 212 Fleckvieh, 127 Brown Swiss and 103 Fleckvieh × Red Holstein dairy cows were evaluated. Although Brown Swiss is a more dairy orientated breed compared with Fleckvieh as dual‐purpose breed, daily milk yield and energy‐corrected milk production were not affected by genotype. Brown Swiss was characterized by a significantly (*p* < 0.05) higher fat and protein content, somatic cell score, and milk urea content. At the same time, Brown Swiss achieved significantly (*p* < 0.05) lower values for dry matter intake and therefore consumed a lower amount of protein and energy per day. Due to these differences, the calculated efficiency parameters for Brown Swiss were significantly higher in the present study, making Brown Swiss more efficient in terms of feed, energy, protein, and metabolic weight. The differences in efficiency can be partly explained by differences in body weight and the associated maintenance requirements. The higher milk urea values combined with lower protein intake of Brown Swiss compared with Fleckvieh under same conditions, confirm current research findings and indicate physiological differences of the Brown Swiss breed. From this perspective, it is worth considering whether the reference values for milk urea should be adjusted according to breed, as milk urea values are indicators of nitrogen supply to rumen microbes, the protein supply status of cows, and the estimation of nitrogen excretion.

## 1 Introduction

Improving efficiency in farm animals has been an ongoing issue (Sauvant 2019) and gained importance in the dairy sector in recent decades. Not only to guarantee a food supply for the world's growing population (Berry and Crowley 2013), but also to improve the profitability of dairy farming, because feed costs account for a large proportion of dairy production costs (Bach 2012; Beever and Doyle 2007; De Haas et al. 2014). Sustainable resource management in agriculture is important not only for economic reasons but also for social and environmental considerations (Boichard and Brochard 2012). Although the agricultural sector has recently made reductions in greenhouse gas (GHG) emissions, it is essential to continue making efforts in this area to achieve international climate targets (Balafoutis et al. 2017). Another challenge of livestock farming is the change in climatic conditions with extreme weather circumstances like heavy rainfall or droughts (Cheng et al. 2022; Godde et al. 2021; Godden et al. 2001; Ledinek et al. 2022). In addition, the available resources for livestock husbandry, such as land, water, and energy, are diminishing due to the advancing urbanization (Boichard and Brochard 2012). In this case, the advantage of ruminants is that they can convert nonedible biomass into food, thus not competing with human nutrition (Windisch 2021).

One component of a common strategy to reduce the GHG regarding dairy production is to increase the milk yield per cow, thereby decreasing the quantity of GHG emitted per kilogram of milk (Zehetmeier et al. 2012). But the increase of milk production per cow is limited because higher milk yield can also lead to health problems in dairy cows (Fleischer et al. 2001; Ingvartsen et al. 2003; Oltenacu and Broom 2010; Opsomer 2015). Nonetheless, milk production per cow increased considerably during the last years, which had a negative impact on cow fertility and vitality and thereby reduced the cows' service lifetime (Knaus 2009).

Efficiency can be defined as a ratio of output to input, an approach that originally comes from economic applications (Ledinek et al. 2022). Efficiency traits can be categorized, for example, according to production factor (input), product (output), calculation period and reference level (animal, farm, production system) (Ledinek et al. 2022). To assess the true efficiency, it is important to consider the changes in body substance, as a mathematically short‐term high efficiency can come at the expense of the animals' health and fertility (Ledinek et al. 2022). Although the cows have been bred to a larger size and a sharper type recently, a smaller body size has a positive effect on efficiency and longevity (Hansen 2000; Vallimont et al. 2011). But there are many factors which can affect efficiency of dairy cows during lactation. Veerkamp and Emmans (1995) presumed that there might be a genetic variation of milk yield, feed intake capacity, the degree of body tissue mobilization and partitioning of the absorbed energy through fodder, which could lead to a genetic variation in energy efficiency. The genetic variation in efficiency is increasingly significant in cattle breeding, with some breeding programs now providing values for efficiency traits (Pryce et al. 2015). The present study tested differences in efficiency of dual‐purpose Fleckvieh (FV), Brown Swiss (BS), and dual‐purpose Fleckvieh × Red Holstein (FV × RHO) dairy cows by assessing the milk production and milk composition traits, feed intake, and body condition traits. Previous studies that examined milk composition traits determined differences in milk urea content (MUC) among breeds. Particularly, the BS breed has frequently been associated with higher MUC values (Doska et al. 2012; Wattiaux et al. 2005). Kessler et al. (2020) previously examined higher milk urea nitrogen (MUN) for BS compared with Holstein (HO) cows under identical conditions. The present study aims to investigate whether the higher MUC of BS can also be demonstrated in comparison to FV under identical housing and management conditions. However, the majority of studies do not provide information on exact feed intake or are only based on estimated values rather than actual measured data. Since MUC is highly dependent on crude protein (CP) intake, it still remains unclear whether the differences arise from variations in feed intake and CP consumption. The present study examined not only MUC but also the measured daily feed intake and thus provides information on the actual protein intake based on a large data set.

## 2 Materials and Methods

The present work is based on 24 feeding trials which took place at the Bavarian State Research Center for Agriculture in Achselschwang, Germany. Data were collected in the period from November 2011 to May 2023. Background of the feeding trials were various questions in cattle feeding and therefore, the herd got separated in two or three feeding groups, depending on the question of the trial and the resulting different nutritional management. Nevertheless, all cows within each trial were kept in the same barn under similar management and environmental conditions. The length of the feeding trials varied between 7 and 20 weeks. Trial weeks > 13, however, were not considered in the analysis, because the majority of the trials ended after 12 weeks (*n* = 15). One trial lasted only 7 weeks, a second one only 9 weeks. The remaining seven trials lasted at least 13 weeks.

### 2.1 Breed Classification

The data consisted of three different breed categories. FV were defined by a minimum proportion of 85% of the FV genotype, which are internationally referred to as German Simmental dual‐purpose cattle. The BS cows were a mixture between the Original Braunvieh and BS with an average of 79% BS genetic. A maximum proportion of 15% of foreign genes (non‐BS or non‐Original Braunvieh) was permitted in BS. As a third group, crossbred cows of FV × RHO with a minimum proportion of 15% Red Holstein Genotype were included. Eleven of the FV × RHO cows had a small proportion of Black Holstein in addition to the Red Holstein proportion. The average FV proportion in this group was 76%, making FV the dominant genotype of the crossbred line.

In the following text, the term ‘breed’ is used to refer to all three genetic groups, even though the crossbred of FV × RHO does not represent a ‘pure’ breed.

### 2.2 Data Collection

The cows were kept in a free stall barn and milked in a milking parlor system twice a day. Daily milk yield (kg/day) was measured based on the two milkings. Each week, an individual milk sample was collected from each cow as a mixture of morning and evening milk and the milk samples were analysed. Fat, protein, lactose, and MUC were analysed by infrared spectroscopy. Somatic Cell Count (SCC) was determined using optical fluorescent techniques. The analysis of the milk samples was conducted in three different laboratories—without laboratory change within a single trial. The proportions of fat (%), protein (%), lactose (%), MUC (mg/L) and SCC (×10^3^ cells/mL) were recorded once a week.

In 18 of the 24 feeding trials, a total mixed ration and in 6 feeding trials, a partial mixed ration combined with a separate concentrate feeding station was fed to the cows. The rations were mixed in a feed mixing wagon (Faresin Pioneer 1600 and Hirl Mertsee SF 1600 HM). The composition of the rations varied depending on the individual trial objective. The mixed rations were provided to the cows ad libitum from special feeding troughs equipped with a weighing unit (Wendl et al. 2001). Each cow wore an ankle tag with an electronic transponder for individual identification purposes. Thus, it was possible to measure the individual feed intake. Samples of the feed ration components and the mixed rations were collected weekly. Each month, a proportionally pooled sample of the weekly samples was analysed. The standard methods according to the Association of German Agricultural Analytic and Research Institutes (Naumann et al. 2012) were applied for the analysis of dry matter (method 3.1), crude ash (method 8.1), crude protein (method 4.1.2), enzyme soluble organic matter (method 6.6.1), gas production (method 25.1), acid detergent fiber (ADFom, method 6.5.2) and neutral detergent fiber (aNDFom, method 6.5.1). Crude fat (method 152‐G) and starch (method 152‐K) were determined according to the methods of the Commission Regulation (EC) No 152/2009 (2009).

The concentrates were tested monthly using the same methods. Energy concentration and utilisable crude protein at the duodenum (uCP) of the feedstuffs was calculated using the equations provided by GfE (2001, 2008) and DLG (2011). Energy and nutrient content of the whole daily ration was calculated from diet composition and energy and nutrient content of single feedstuffs. Based on the analyses, the dry matter intake (DMI in kg/day) and intake of energy (net energy for lactation [NEL] in MJ/day; metabolizable energy [ME] in MJ/day according to GfE 2001), as well as the daily intake of CP (g/day) and uCP (g/day) were calculated by multiplying the DMI with the analysed nutrient contents. The requirements and supply of energy and nutrients were calculated according to the guidelines of GfE (2001).

The individual body weight (BW) of each cow was recorded at the beginning, in the middle, and at the end of each trial using an electronic scale (Taxatron 5000; GEA, accuracy: 1% from 0 to 1000 kg weight). At the same time, the body condition score (BCS) on a scale from 1 (lean) to 5 (obese) and the back fat thickness (BFT) in centimetres were determined using an ultrasound scanner (Tringa Linear Vet; Esaote Europe BV). The method of Staufenbiel (1992) was used for BFT recordings. BCS was assessed simultaneously by two raters and scored according to Edmonson et al. (1989) and Jilg and Weinberg (1998). The three values of the beginning, middle, and end of the trial for BW, BCS an BFT were used for linear interpolation to estimate the daily values for each cow.

The final data set consisted of 442 different cows, with some cows participating in more than one trial. A total of 99,288 daily records entered the data set (Table 1).

**Table 1:** Descriptive statistics of the number of cows, daily records, average days in milk (DIM) and parity for Fleckvieh, Brown Swiss and Fleckvieh × Red Holstein.

Item	Fleckvieh (FV)	Brown Swiss (BS)	Fleckvieh × Red Holstein (FV × RHO)	Total
Cows (*n*)	212	127	103	442
Daily records (*n*)	43,770	31,612	23,906	99,288
Average DIM (days)^a^	168 ± 60	167 ± 62	169 ± 61	
Average parity (number of parity)^a^	3.04 ± 1.9	3.28 ± 2.0	3.45 ± 2.2	

a Mean ± SD.

### 2.3 Trait Calculations

Energy‐corrected milk (ECM) was calculated as follows: ECM (kg) = milk yield (kg) × [0,38 × (fat %) + 0,21 × (protein %) + 1,05]/3,28 according to GfE (2001). The SCC was converted to somatic cell score (SCS) using the formula SCS = log2 (SCC/100.000) + 3 (Ali and Shook 1980). The energy content of the milk (LE) was calculated according to GfE (2001) as follows LE (MJ/kg) = 0.38% fat + 0.21. % Protein + 0,95, while the metabolic body weight was determined as BW^0.75^. MUN was calculated by multiplying the MUC (mg/dL) by 0.467 (Kessler et al. 2020).

The energy balance was calculated according to Flachowsky et al. (2009) and GfE (2001). The calculation consisted of the energy intake from the feed (MJ NEL), from which the maintenance requirement (MJ NEL/kg BW^0.75^ and day) and the energy output through milk (MJ/kg milk), as well as an energy allocation for the growth of calf and uterus (MJ NEL/day) starting from the 6th week before calving, were subtracted.

### 2.4 Efficiency Parameters

The calculations of efficiency parameters were based on the descriptions of Ledinek et al. (2022). Feed efficiency was defined as kg ECM/kg DMI. Energy efficiency was calculated as kg ECM/10 MJ NEL and milk energy efficiency as MJ LE/10 MJ NEL. For protein efficiency, these three ratios were used: g milk protein/kg dry matter, g milk protein/10 MJ NEL, and kg ECM/kg uCP. As a further efficiency parameter, metabolic body weight efficiency was determined as kg ECM/kg^0.75^ metabolic body weight.

### 2.5 Data Editing and Statistical Analysis

All observations of normally distributed parameters, that exceeded ± 4.0 SD from the mean were eliminated. This included the records of milk yield, ECM, milk ingredients, SCS, feed intake, BW, and BFT. All observations of the BCS from 1 to 5 were included. The check of normality for the individual variables was conducted by comparing a histogram of the sample data to a normal probability curve. In addition, a quantile‐quantile plot (QQ plot) of the standardized data against the standard normal distribution was used as a graphical aid for assessing normality by using SAS/IML Studio 14.2 (SAS Institute Inc.). Days in milk (DIM) were restricted from 40 to 305 days. The reason for this limit is, that within the data set, the under 40‐DIM range did not represent all three breeds. Moreover, the DIM were divided into 13 lactation periods, with each period comprising 20 DIM. The lactations 1–5 were considered individually, but all cows within lactation 6 and above were grouped in one lactation category. The feeding trials were spread over the whole year, so they took place in different months and therefore in different seasons. For that reason, 2 consecutive months starting with January were combined to one double‐month, resulting in six seasonal categories. Four gestation periods were considered: nonpregnant and the first, second and third trimester of gestation.

SAS 9.4 (SAS Institute) was used for the statistical analysis. An analysis of variance (ANOVA) with a MIXED procedure by using a Restricted Maximum Likelihood Analysis (REML) was applied (SAS Institute Inc. 2015. SAS/STAT® 14.1 User's Guide). The fixed effects included in the statistical model were breed, feeding trial, lactation, lactation period, gestation period, season, as well as the interaction effects breed × feeding trial, breed × lactation, breed × lactation period, breed × gestation period, breed × season, and feeding group within feeding trial. For the analysis of MUC according to protein intake classes, the interaction effect of breed × protein intake class was additionally included in the model.

As some cows were present in more than one trial, the individual cow was always included as a random effect.

The differences between the three breeds and among the other fixed effects were tested using the Tukey *t* test, with a significance level set at *p* < 0.05. In addition to the F test results, all results are shown as least squares means (LSM) ± standard errors of means (SEM).

## 3 Results and Discussion

As shown in Table 1, no major differences were found between the breeds in terms of DIM and parity, which may have affected the results of the subsequent variance analysis.

### 3.1 Feed Intake

Differences in DMI could be attributed to various influencing factors, including genetic origins. Berry and Crowley (2013) cited differences in BW, growth rate, milk yield, body composition, body size and muscularity as reasons for the genetic variation in feed intake. It is described that breeds focused on milk show a higher DMI (0.5–1.0 kg/day) compared with other breeds, which is partly due to the larger frame of the animals (GfE 2023; Gruber et al. 2004).

In the present study, daily DMI of BS was 1 and 0.7 kg lower compared with FV and FV × RHO, respectively (*p* < 0.05, Table 2). As a result, the intake of ME and NEL was significantly lower for BS compared with the other two breeds (*p* < 0.05). Daily intake of CP and uCP was 134 and 137 g/day lower (*p* < 0.05, Table 2) in BS compared with FV, whereas FV × RHO lay in between the other breeds. However, the lower DMI of BS could be explained by the lower BW (Table 4). With an additional 100 kg BW, DMI can increase by between 0.8 and 1.3 kg depending on the stage of lactation (GfE 2023). In contrast, Ledinek et al. (2019) reported higher DMI for FV × RHO with 25% RHO proportion compared with BS and FV (*p* < 0.05) but the DMI of the pure breeds was comparable. However, results of the aforementioned study were based on predicted feed intake but not on measured values.

**Table 2:** Comparison of daily feed intake and daily intake of protein and energy for Fleckvieh (FV), Brown Swiss (BS) and Fleckvieh × Red Holstein (FV × RHO) dairy cows kept under same conditions (LSM ± SEM).

Breed	FV	BS	FV × RHO	*p* value (breed)
DMI (kg/day)	23.5 ± 0.1^a^	22.5 ± 0.2^b^	23.2 ± 0.2^a^	0.0002
Roughage dry matter (kg/day)	15.2 ± 0.1^a^	14.5 ± 0.1^b^	15.1 ± 0.1^a^	< 0.0001
Concentrate dry matter (kg/day)	8.30 ± 0.1^a^	7.92 ± 0.1^b^	8.07 ± 0.1^ab^	0.0123
CP (g/day)	3526 ± 23^a^	3392 ± 30^b^	3492 ± 33^ab^	0.002
uCP (g/day)	3622 ± 23^a^	3485 ± 30^b^	3587 ± 33^ab^	0.0014
RNB (g/day)	−15.5 ± 0.3	−14.7 ± 0.4	−15.0 ± 0.4	0.2838
ME (MJ/day)	267 ± 1.7^a^	256 ± 2.2^b^	264 ± 2.4^a^	0.0009
NEL (MJ/day)	163 ± 1.0^a^	156 ± 1.3^b^	161 ± 1.5^a^	0.001

Abbreviations: CP, crude protein (GfE 2001); DMI, dry matter intake; ME, metabolizable energy (GfE 2001); NEL, net energy for lactation (GfE 2001); RNB, ruminal nitrogen balance; uCP, utilisable crude protein at the duodenum (GfE 2001).

^a,b^Least squares means with different superscripts within rows are significantly different (*p* < 0.05).

Recent analyses by Gruber et al. (2021) showed that FV and HO can reach the same feed intake capacity when animal‐ and feed‐related factors, such as lactation number, lactation stage, body mass, milk yield, concentrate level, and forage quality, are held constant. This is consistent with the present results because the crossbreeding of HO into FV does not result in any difference in feed intake (*p* < 0.05, Table 2). The increase in milk production in dual‐purpose breeds like FV has led these animals to become more similar to high‐performance breeds like HO (Gruber et al. 2021).

To summarize, BS had lower DMI, protein and energy intake even tough BS belongs to the more specialized dairy breeds and therefore it could be expected that BS cows have higher intakes compared with more beef‐oriented breeds like FV (GfE 2023).

### 3.2 Milk Production and Milk Composition Traits

FV is known as a dual‐purpose breed for both milk and meat production (ASR 2024a; Rinderzuchtverband Oberpfalz 2022; Weser‐Ems‐Union eG 2020). BS is considered as a dual‐purpose breed, with an increased emphasis on milk (ASR 2024b). A comparison between FV and HO shows that the higher milk yield of more dairy‐oriented breeds is compensated by advantages in other areas like energy balance, economic returns from old cows and calves or the CO_2_ footprint and resource efficiency when meat and milk are included in the consideration (Spiekers et al. 2022). Despite the more dairy‐oriented focus of BS, FV outperformed BS in the Bavarian averages in 2023 in terms of milk production (LKV 2023).

The present study failed to show differences among the three breeds for daily milk yield and ECM (*p* > 0.05, Table 3). Ledinek et al. (2019) presented that the introgression of a proportion of 25% HO into FV resulted in a significantly (*p* < 0.05) higher milk yield compared with FV. This increase in performance due to crossbreeding could not be confirmed in the present trials (Table 3). Regarding milk production traits, BS and FV exhibited comparable results. These findings are consistent with those reported by Ledinek et al. (2019), Penasa et al. (2014), Bobbo et al. (2014), Benedet et al. (2020), and Gottardo et al. (2017). This could imply, that the FV breed has caught up with, if not surpassed, BS in terms of milk production. A possible explanation could be the larger population of FV and the resulting faster breeding progress.

**Table 3:** Comparison of daily milk production and milk composition traits for Fleckvieh (FV), Brown Swiss (BS), and Fleckvieh × Red Holstein (FV × RHO) kept under the same conditions (LSM ± SEM).

Breed	FV	BS	FV × RHO	*p* value (breed)
Milk yield (kg/day)	33.4 ± 0.3	33.5 ± 0.4	34.1 ± 0.5	0.5038
ECM (kg/day)	33.3 ± 0.3	34.3 ± 0.4	33.8 ± 0.4	0.1051
Fat (%)	3.91 ± 0.03^b^	4.09 ± 0.03^a^	3.88 ± 0.04^b^	< 0.0001
Protein (%)	3.62 ± 0.01^b^	3.71 ± 0.02^a^	3.61 ± 0.02^b^	< 0.0001
Lactose (%)	4.73 ± 0.009	4.72 ± 0.011	4.71 ± 0.013	0.6704
Milk fat (kg/day)	1.28 ± 0.01^b^	1.35 ± 0.02^a^	1.30 ± 0.02^ab^	0.0022
Milk protein (kg/day)	1.20 ± 0.01	1.23 ± 0.01	1.21 ± 0.02	0.1375
MUC (mg/L)	192 ± 1.9^b^	204 ± 2.4^a^	184 ± 2.7^b^	< 0.0001
MUN (mg/dL)	8.95 ± 0.09^b^	9.53 ± 0.11^a^	8.60 ± 0.13^b^	< 0.0001
Somatic Cell Score (SCS)	2.69 ± 0.07^b^	2.97 ± 0.09^a^	2.62 ± 0.10^b^	0.0168

Abbreviations: ECM, energy‐corrected milk; MUC, milk urea content; MUN, milk urea nitrogen content.

^a,b^Least squares means with different superscripts within rows are significantly different (*p* < 0.05).

BS is known for their good coagulation properties of milk, high protein quality and their greater fat and protein content (ASR 2024b; De Marchi et al. 2007). In the context of the present study, BS showed about 0.2% and 0.1% higher milk fat and protein content (*p* < 0.05) than FV and FV × RHO, whereas all three breeds had similar milk lactose contents (Table 3). Nevertheless, differences in the absolute daily amount of produced protein (kg) in milk were absent, but the absolute daily amount of fat (kg) was around 0.07 kg higher (*p* < 0.05) for BS compared with FV. Gottardo et al. (2017), Benedet et al. (2020) and Penasa et al. (2014) achieved similar results comparing milk compositions of BS and FV, with BS showing a significant higher fat and protein content. In contrast, Ledinek et al. (2019) predicted no differences in protein content between BS and FV. De Marchi et al. (2007) only confirms the present results concerning the higher protein content of BS but could not detect any differences in fat content comparing BS and FV. Previous reports showed the superiority of BS in fat and protein content compared with other dairy breeds like HO in multibreed herds (Bobbo et al. 2014; De Marchi et al. 2008; Kessler et al. 2020, 2024) and under same conditions (Kessler et al. 2020, 2024). The present results are therefore consistent with a large number of studies that have shown that BS has a special milk composition in terms of quality characteristics.

In general, MUC and MUN reached lower values in our study compared with other studies and were slightly below the target range of 10 to 16 mg/dL for MUN (Jonker et al. 1999) and in the lower to middle range for MUC (150–250 mg/L) (Glatz‐Hoppe et al. 2020; Guliński et al. 2016). The values of MUC and MUN can exhibit significant variation and are affected by various factors (e.g., nutrition, season, parity, DIM and breed) (Doska et al. 2012; Rajala‐Schultz and Saville 2003). The precise planning and implementation of the rations in the trials, as well as a general effort to reduce protein excess in dairy cow feeding in recent years, could be a possible explanation for the lower values.

In the present analysis, MUC and MUN were significantly (*p* < 0.05) higher in BS cows than in FV and FV × RHO. These results were similar to those presented by Benedet et al. (2020) and Gottardo et al. (2017), who reported significantly (*p* < 0.05) higher MUC and MUN in BS compared with FV. BS consumed less CP than FV and FV × RHO (Table 2) while achieving higher MUC and MUN. This is worth mentioning because it has been described that MUN correlates positively with dietary protein intake (Spek et al. 2013). Accordingly, lower values for MUC and MUN would have been expected due to a lower protein intake.

**Figure 1:**
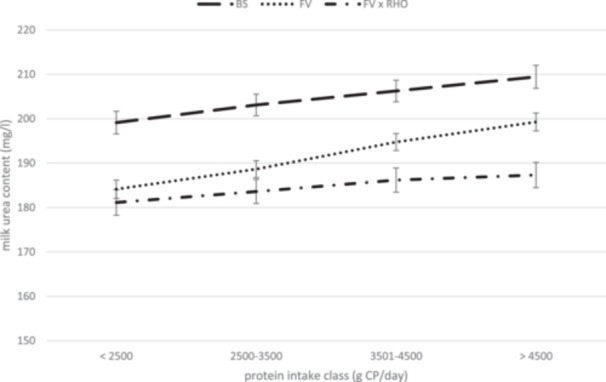
Milk urea content presented by protein intake class of Fleckvieh (FV), Brown Swiss (BS) and Fleckvieh × Red Holstein (FV × RHO) dairy cows (LSM ± SEM, *p* < 0.0001 for interaction effect breed × protein intake class).

Figure 1 displays the values for MUC categorized by protein intake class. Four classes were established based on the CP intake of the cows. BS consistently achieved higher MUC values across all four CP intake classes. This further highlights the elevated MUC values of BS, regardless of the CP intake.

There have been several studies showing higher MUN for BS compared with HO (Doska et al. 2012; Gruber et al. 2014; Wattiaux et al. 2005). These results can also be achieved under the same feeding and management conditions (Kessler et al. 2020). In this study, we were able to confirm the superiority of BS in MUC and MUN compared with FV under same conditions. Because the cows were kept under same conditions, this suggests a breed‐specific difference to BS. At present, the reasons of these differences in MUN remain unclear and can only be speculated, but genotypic differences in protein metabolism like urea production and excretion were discussed (Kessler et al. 2020). Kessler et al. (2024) took the kidney function into account. Their results showed greater concentrations of urea, creatinine and SDMA (symmetric dimethylarginine) in blood samples of BS compared with HO, while urea and creatinine concentrations in urine and saliva were not affected by breed. Because SDMA is known and used as a biomarker for determining the glomerular filtration rate (GFR), these results indicate a lower GFR for BS (Kessler et al. 2024). Breed‐specific differences in GFR could be responsible for the higher MUN and MUC values in this study.

Benedet et al. (2020) analysed blood urea nitrogen (BUN) and found the highest values for BS cows compared with HO and FV in multibreed herds. Because MUN is high correlated with BUN (Broderick and Clayton 1997; Hof et al. 1997), these blood tests confirm the results of the present study and the results of Kessler et al. (2024).

An environmental issue of livestock farming is the nitrogen (N) pollution in form of N compounds like emitting ammonia into the atmosphere and nitrate into surface and groundwater (Draaijers et al. 1989; Howarth et al. 1996). MUC is correlated to the excretion of N (Jahnel et al. 2021) and is often used as a tool to estimate the amount of N excretion and the protein supply to the cow through feed (Nousiainen et al. 2004). It needs to be considered whether a breed‐specific target range for MUN and MUC is required, as current studies indicate that BS has physiologically higher MUC values. Possible variations in urinary urea excretion due to physiological characteristics need to be further investigated. Kessler et al. (2024) already showed that there were no differences in faecal nitrogen content between BS and HO.

The risk of mastitis is moderately to strongly correlated with SCS (*r* = 0.30–0.80) (Franzoi et al. 2020). A high SCC and SCS, respectively, is known, since the 1960s, as a first indicator of inflammation caused by a migration of neutrophils (Arnould et al. 2013). In the present study, SCS was notably higher for BS than for FV and FV × RHO (Table 3). The findings of Gottardo et al. (2017), Benedet et al. (2020) and De Marchi et al. (2007) confirm the present results. On the other hand, Penasa et al. (2014) observed no differences in SCS between BS and FV. The question remains open whether BS had poorer udder health and thus a higher susceptibility to mastitis due to the higher SCS values.

### 3.3 Body Condition

One difficulty in assessing the efficiency of dairy cows—in combination with normal variations in body condition—is their ability to mobilize body fat and the usage of body tissue energy for milk production (Berry and Crowley 2013; Roche et al. 2009). These processes can give the false impression of high efficiency, if the cow extensively mobilizes a considerable amount of body tissue at the beginning of lactation and the BCS consequently decreases (Köck et al. 2018). Reliable information on BCS, fertility and health is therefore crucial for assessing efficiency (Köck et al. 2018).

FV and FV × RHO were almost identical in terms of BW, BCS and RFD (Table 4). BS weighed less, had a lower BCS and a lower BFT compared with the other two breeds (Table 4). This was expected, as BS is a breed that is more focused on milk production and should therefore have a lower BW and BCS than a dual‐purpose breed such as FV (Köck et al. 2018; Ledinek et al. 2019).

**Table 4:** Body weight, back fat thickness and body condition score for Fleckvieh (FV), Brown Swiss (BS) and Fleckvieh × Red Holstein (FV × RHO) dairy cows (LSM ± SEM).

Breed	FV	BS	FV × RHO	*p* value (breed)
Body weight (kg)	783 ± 5^a^	709 ± 6^b^	775 ± 7^a^	< 0.0001
BFT (cm)	2.13 ± 0.05^a^	1.82 ± 0.07^b^	2.05 ± 0.07^ab^	0.0011
BCS	3.97 ± 0.04^a^	3.55 ± 0.05^b^	3.84 ± 0.06^a^	< 0.0001

Abbreviations: BCS, body condition score; BFT, back fat thickness.

^a,b^Least squares means with different superscripts within rows are significantly different (*p* < 0.05).

**Figure 2:**
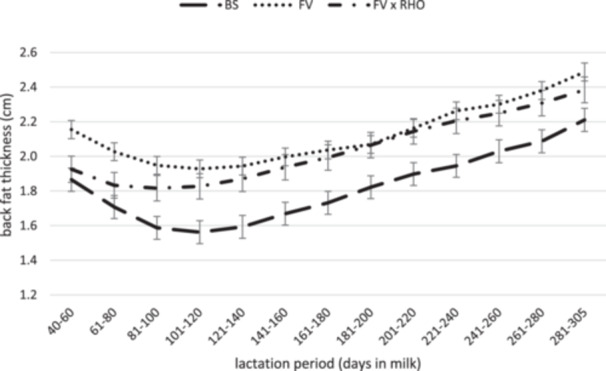
Back fat thickness throughout the lactation from 40 to 305 days in milk (DIM) of Fleckvieh (FV), Brown Swiss (BS) and Fleckvieh × Red Holstein (FV × RHO) dairy cows (LSM ± SEM, *p* < 0.0001 for interaction effect breed × lactation period).

**Figure 3:**
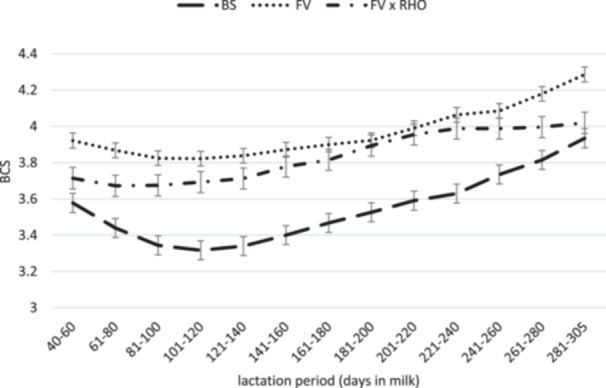
Body condition score throughout the lactation from 40 to 305 days in milk (DIM) of Fleckvieh (FV), Brown Swiss (BS) and Fleckvieh × Red Holstein (FV × RHO) dairy cows (LSM ± SEM, *p* < 0.0001 for interaction effect breed × lactation period).

In Figures 2 and 3, the course of BCS and BFT from 40 to 305 DIM of the three breeds is shown. The curves mirror the course during lactation divided into sections of 20 lactation days. For all three groups, the course of the curves was typical for dairy cows. Approximately 40–100 days postpartum, cows reduce body substance (mainly body fat) to subsequently refill the energy reserves until the next calving (Roche et al. 2009). Catabolizing and replacing body tissue represents an additional energy expenditure for the cow (Coffey et al. 2002). FV and BS had their lowest BCS in the period between 101 and 120 DIM, whereas FV × RHO already reached their lowest point in the period between 61 and 80 DIM and thus began to rebuild body reserves earlier in lactation. After a rather small decline, the BCS curve of FV × RHO runs relatively straight upwards compared with the other breeds. This suggests that FV × RHO mobilized less body tissue during early lactation and rebuilt tissue more slowly towards the end of lactation. Therefore, the question arises whether crossbred animals have a lower susceptibility to metabolic diseases.

### 3.4 Efficiency Parameters

The comparison of the BFT curves between BS and FV shows that the progression is approximately the same. The difference in efficiency is therefore probably not due to the changes in body fat reserves. The BCS curve showed a slightly more pronounced decline for BS in comparison to FV during early lactation. Whether this resulted in an increased loss of muscle mass cannot be precisely determined based on the figures.

The efficiency of the crossbred animals FV × RHO is probably somewhat underestimated, as a stronger energy accumulation can be observed compared with BV and FV. However, all efficiency parameters showed comparable results between FV and FV × RHO (*p* > 0.05, Table 5). It is noticeable that BS had significantly higher values of efficiency for feed, energy, protein, and metabolic body weight compared with FV and FV × RHO (*p* < 0.05). In contrast to the results reported by Ledinek et al. (2019), who only indicated a higher body weight efficiency without any observed advantages in feed and energy efficiency for BS. But the analyses of (Ledinek et al. 2019) were partially based on estimated and not on measured values. Köck et al. (2018) also obtained the same feed efficiency (ECM/DMI) for BS and FV using estimated values for feed intake. Even if there is a high degree of agreement between values measured and estimated in the literature, this could be a reason for the differences between the studies.

**Table 5:** Comparison of calculated efficiency traits for energy, feed, protein and metabolic body weight for Fleckvieh (FV), Brown Swiss (BS) and Fleckvieh × Red Holstein (FV × RHO) dairy cows (LSM ± SEM).

Breed	FV	BS	FV × RHO	*p* value (breed)
Feed efficiency (kg ECM/kg dry matter)	1.44 ± 0.01^b^	1.58 ± 0.02^a^	1.48 ± 0.02^b^	< 0.0001
Energy efficiency (kg ECM/10 MJ NEL)	2.08 ± 0.02^b^	2.27 ± 0.02^a^	2.13 ± 0.02^b^	< 0.0001
Energy efficiency (LE in MJ/10 MJ NEL)	6.60 ± 0.05^b^	7.23 ± 0.07^a^	6.77 ± 0.08^b^	< 0.0001
Protein efficiency (g milk protein/kg dry matter)	51.7 ± 0.4^b^	56.7 ± 0.6^a^	53.3 ± 0.6^b^	< 0.0001
Protein efficiency (g milk protein/10 MJ NEL)	74.6 ± 0.6^b^	81.6 ± 0.8^a^	76.5 ± 0.9^b^	< 0.0001
Protein efficiency (kg ECM/kg uCP)	9.34 ± 0.1^b^	10.2 ± 0.1^a^	9.59 ± 0.1^b^	< 0.0001
Metabolic body weight efficiency (kg ECM/kg^0.75^ metabolic body weight)	0.23 ± 0.002^b^	0.25 ± 0.003^a^	0.23 ± 0.003^b^	< 0.0001

Abbreviation: LE, energy in milk.

^a,b^Least squares means with different superscripts within rows are significantly different (*p* < 0.05).

In our study, the superiority of BS in terms of efficiency is based on the higher milk fat and protein contents combined with the lower feed intake and the reduced BW. Low feed intake positively affects efficiency because the input is reduced. On the other hand, lower feed intake in dairy cows must be evaluated very critically, because feed intake increases more slowly than milk production in early lactation (Coffey et al. 2004). This time lag leads to a period when the required energy cannot be covered by the feed intake and therefore the energy must be mobilized from body reserves (Coffey et al. 2004). An extreme negative energy balance (NEB) especially in early lactation, can lead to diverse health issues such as metabolic diseases, fertility disorders or locomotive problems (Collard et al. 2000). A low feed intake requires very critical evaluation and should not be considered positive per se, even if it may improve feed efficiency. Focusing on lower DMI in selection could heighten the risk of diseases, especially in the early stages of lactation (Becker et al. 2022).

**Figure 4:**
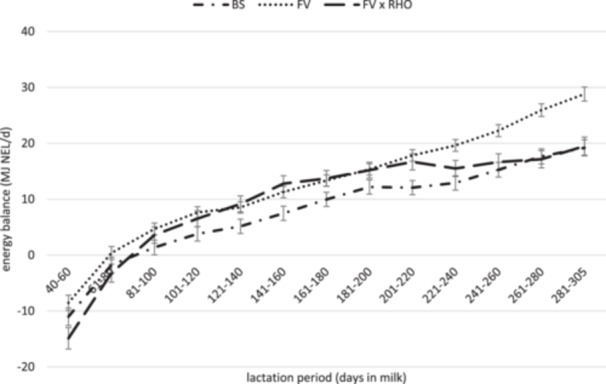
Calculated energy balance of Fleckvieh (FV), Brown Swiss (BS) and Fleckvieh × Red Holstein (FV × RHO) dairy cows during lactation (LSM ± SEM).

In the present study, it was possible to calculate an energy balance that accounts for energy intake from feed, energy output through milk, and the requirements for maintenance and gestation (Flachowsky et al. 2009). As shown in Figure 4, this analysis demonstrates that FV achieved a positive energy balance slightly earlier than the two other breeds. Additionally, the energy balance of BS remained below the curve of FV throughout the entire period. These disparities may indicate that BS needed to mobilize more body reserves in comparison to FV during lactation and especially in early lactation. Compared with HO, FV was also able to achieve a positive energy balance earlier in lactation, while exhibiting lower energy efficiency (Ledinek et al. 2023; Spiekers et al. 2022). On the other hand, the studies of Benedet et al. 2020 and Urdl et al. 2015 showed similar NEFA (nonesterified fatty acids) concentrations in early lactation for FV and BS. The findings of Benedet et al. (2020) suggested greater BHB (β‐hydroxybutyrate) concentrations for FV compared with BS, whereas Urdl et al. (2015) reported significantly lower BHB concentrations for FV. NEFA and BHB are commonly considered as blood parameters associated with energy metabolism for assessing energy balance, which is why these results partially contrast with those of the present study (Quiroz‐Rocha et al. 2010; Urdl et al. 2015).

However, it should be noted, that the values for energy balance in the present study were only simplified balanced values and no actual measured values. Not all factors that affect the energy balance can be considered in this calculated analysis. For a realistic assessment of the energy balance, changes in the body constitution, such as the anabolism and catabolism of body tissue, would need to be taken into account. Furthermore, the early period of lactation (1–40 DIM) was not included in the present analyses at all. And if a higher proportion of the maintenance requirement was assumed (see further down in the text [GfE 2023]), the calculated energy balance would also change, because FV would consequently have a greater maintenance requirement.

To accurately measure and quantify the negative energy status, further tests should be conducted, ideally covering the entire lactation, including the dry period.

Breeding for higher milk yield has consistently resulted in larger and heavier animals (Hansen 2000). But this trend does not necessarily lead to better efficiency. Vallimont et al. (2011) found that smaller cows with lower BW are more efficient, which is attributed to a negative correlation between BCS and BW in relation to efficiency traits. Calculations by Gruber (2013) revealed that for every additional 100 kg of weight, a cow must produce an average of 844 kg (802–894 kg) more milk per lactation to maintain the same energy efficiency. One reason of the differences in efficiency in this study could be the discrepancy in maintenance requirements due to the different BW. Calculated according to GfE (2001) and using the BW given in Table 4, the daily maintenance requirement of the three genetics was 43.4, 40.3, and 43.0 MJ NEL for FV, BS, and FV × RHO, respectively. Based on the new calculations according to GfE (2023), the maintenance requirement was higher (FV 94.7 MJ ME, BS 87.9 MJ ME, FV × RHO 94.0 MJ ME). The differences in energy intake between FV and BS were 7.0 MJ NEL and 11.0 MJ ME (Table 2). Accordingly, 44% (GfE 2001) and 62% (GfE 2023) of the differences in energy intake can be explained by the different maintenance requirements. It is therefore assumed that the maintenance requirements of high‐performance animals are higher than previously estimated (Gruber et al. 2021). This can be attributed to an increased metabolic demand for higher milk production and a greater weight of internal organs (Gruber et al. 2021).

The breed differences in weight and the associated differences in maintenance requirements could partially explain the significantly higher efficiency parameters for BS. Depending on how precisely the maintenance requirement is calculated or recorded, a varying proportion of these differences can be attributed to differences in maintenance needs. A limitation of the present study that should be mentioned is the weight measurement and the BCS and BFT assessment, which was only conducted three times per trial (at the beginning, middle, and end). The remaining values per day were interpolated. Thus, the data on BW, BCS and BFT do not align exactly with the feeding data.

In the present study, the focus is on dairy production. However, a holistic approach is required to measure and compare efficiency. The male offspring must therefore also be taken into account. As FV is a dual‐purpose breed, FV has advantages in terms of meat production and the fattening performance of the bulls or heifers (Spiekers et al. 2022). Compared with BS, FV bulls achieve higher daily weight gains, a lower energy expenditure per kg growth, a more favourable carcass yield and carcass classification, as well as a lower fat content, while BS bulls can also achieve high fattening performance (Ettle et al. 2018).

The fertility of the cows is an additional factor that must be considered in the evaluation of efficiency among breeds. Studies indicate that there are breed‐related differences in fertility with dual‐purpose breeds demonstrating greater fertility compared with more milk‐oriented breeds (Bittante et al. 2020; Toledo‐Alvarado et al. 2017). This observation is also supported by studies that investigate the reproductive ability by specific fertility traits. BS cows exhibited average fertility traits that were intermediate between FV and HO, leaning more towards HO (Martinez‐Castillero et al. 2020). This indicates, that FV has a better reproductive capacity then BS (Toledo‐Alvarado et al. 2017). However, only minimal differences were determined in the nonreturn rate on the 56th and 90th day after the first insemination between FV and BS (LKV 2023). In the present study, we were unable to compare fertility, as the evaluated trials were not designed for that purpose.

For a comprehensive assessment of efficiency, other factors such as service lifetime and age at first calving are also relevant. However, these exceed the scope of this study.

Evaluating efficiency on the basis of calculated parameters has the disadvantage that the perspective is very one‐sided, because it leads to maximize the output of one production factor and does not account for advantages or disadvantages that arise elsewhere (Ledinek et al. 2021). Important issues like animal health, should also be evaluated and included in the assessment of efficiency. Further investigations are needed to determine whether BS are more vulnerable to diseases and whether there is a connection to the higher efficiency. Selection of highly efficient cows may lead to a higher susceptibility to diseases. In recent years, dairy cattle breeding has increasingly emphasized health, fertility, and longevity. It is essential to ensure that the focus on efficiency does not undermine this positive trend and that these important health traits are given highest priority (Becker et al. 2021).

## 4 Conclusions

It is concluded that under identical housing and management conditions, BS showed significant differences regarding the milk composition traits by achieving greater milk protein and fat content, SCS and MUC. As milk production was not affected by genotype, the question arises as to what extent BS is still more dairy‐oriented than FV, or whether breeding has already altered this difference.

Compared with FV and FV × RHO, BS had a reduced DMI and lower protein‐ and energy intake at identical diet composition. As a result, the calculated efficiency parameters (feed, energy, and protein efficiency, as well as metabolic body mass efficiency) were significantly higher in BS, making BS considerably more efficient in the present calculations. Part of the difference can be explained by the higher need for maintenance due to differences in BW. The extent to which the superiority of BS in efficiency resulted from changes in body composition and energy balance could not be conclusively determined. However, there are indications that differences do exist in this regard, as FV achieved a positive energy balance earlier in lactation.

The higher MUC and MUN values and the simultaneously lower protein intake support the assumption that BS exhibits physiological specifies in urea excretion. It needs to be considered whether a breed‐specific target range for MUC is required, because it is common practice to estimate N excretion, and the protein supply status of cows based on the MUC.

The crossbreed of FV × RHO with an RHO proportion of approximately 25%, which is commonly found in practical farms, showed no differences in efficiency compared with pure FV cows. This leads to the conclusion, that the Holstein characteristics of the crossbred animals were not strongly pronounced. FV × RHO mobilized less body tissue in early lactation and rebuilt tissue more slowly towards the end of lactation. This suggests that FV × RHO can cope better with metabolic stress.

## Ethics Statement

The experiments were conducted at the Bavarian State Research Center for Agriculture (Bayerische Landesanstalt für Landwirtschaft, LfL) according to European guidelines for animal experiments (Directive 2010/63/EU, 2010) and were approved by the ethics committee of the Ethics of Animal Experiments of LfL.

## Conflicts of Interest

The authors declare no conflicts of interest.

## Supporting information

Supporting_information_for_online_publication_only.

## Data Availability

The data that support the findings of this study are available from the corresponding author upon reasonable request.
