# Supplementary material for: Comparative Study on Milk Production, Milk Components, Feed Intake and Efficiency Parameters of Fleckvieh (German Simmental), Brown Swiss and Fleckvieh × Red Holstein Dairy Cows
Source: J Anim Physiol Anim Nutr (Berl). 2025 May 19;109(5):1161–73. doi: 10.1111/jpn.14124 (PMC12451417; doi:10.1111/jpn.14124)
Supplement: Supplementary file 1 — Supporting_information_for_online_publication_only. [file JPN-109-1161-s001.docx]

**Supporting Information**

Figure 1 Data structure used by feeding trial and length of the trial in weeks

Figure 2 Data structure limited to days in milk between 40 and 305 by trial

Table 1 Number and subject of feeding trials

| Number of feeding trial | Research topic of the feeding trial |
| --- | --- |
| Feeding trial 1 | Use of soy extraction meal and rapeseed extraction meal in dairy cow feeding |
| Feeding trial 2 | use of soy extraction meal and rapeseed extraction meal in dairy cow feeding (second trial) |
| Feeding trial 3 | use of grass pellets in dairy cow feeding |
| Feeding trial 4 | use of fermented wheat in dairy cow feeding |
| Feeding trial 5 | use of living yeast in dairy cow feeding |
| Feeding trial 6 | comparison of two silage maize varieties and effects on feed intake and milk yield |
| Feeding trial 7 | influence of concentrate feed level on feed intake and milk yield |
| Feeding trial 8 | influence of structural supply through variation of roughage and concentrate feed levels |
| Feeding trial 9 | use of corn shredlage (first study) |
| Feeding trial 10 | use of a compact total mixed ration by using a wet grass silage and adding water |
| Feeding trial 11 | use of a compact total mixed ration by using a normal dry grass silage and adding water |
| Feeding trial 12 | use of corn shredlage (second study) |
| Feeding trial 13 | addition of straw to a partial mixed ration |
| Feeding trial 14 | supply of amino acids, use of rumen-protected methionine |
| Feeding trial 15 | supply of amino acids, use of rumen-protected methionine |
| Feeding trial 16 | supply of amino acids, use of rumen-protected methionine and lysine |
| Feeding trial 17 | Effects of crude protein oversupply in diets of high-yielding dairy cows on performance criteria |
| Feeding trial 18 | supply of amino acids, use of rumen-protected histidine |
| Feeding trial 19 | supply of amino acids, use of rumen-protected methionine |
| Feeding trial 20 | addition of water to a total mixed ration |
| Feeding trial 21 | effects of an increased crude protein supply on performance criteria and body condition on dairy cows in late lactation |
| Feeding trial 22 | use of feeding coal |
| Feeding trial 23 | influence of phosphorus-adapted feeding on performance and feed intake |
| Feeding trial 24 | use of rumen-protected rapeseed meal |

Table 2 Distribution of breeds and total number of cows within the individual trials

|  | Brown Swiss (n cows) | Fleckvieh (n cows) | Fleckvieh x Red Holstein (n cows) | Total (n cows) |
| --- | --- | --- | --- | --- |
| Number of feeding trial |  |  |  |  |
| Feeding trial 1 | 18 | 16 | 14 | 48 |
| Feeding trial 2 | 11 | 21 | 16 | 48 |
| Feeding trial 3 | 18 | 14 | 16 | 48 |
| Feeding trial 4 | 16 | 21 | 11 | 48 |
| Feeding trial 5 | 16 | 17 | 15 | 48 |
| Feeding trial 6 | 12 | 20 | 14 | 46 |
| Feeding trial 7 | 16 | 18 | 14 | 48 |
| Feeding trial 8 | 20 | 14 | 14 | 48 |
| Feeding trial 9 | 16 | 17 | 15 | 48 |
| Feeding trial 10 | 17 | 13 | 13 | 43 |
| Feeding trial 11 | 16 | 18 | 14 | 48 |
| Feeding trial 12 | 20 | 20 | 8 | 48 |
| Feeding trial 13 | 18 | 16 | 14 | 48 |
| Feeding trial 14 | 15 | 22 | 10 | 47 |
| Feeding trial 15 | 17 | 17 | 12 | 46 |
| Feeding trial 16 | 10 | 22 | 14 | 46 |
| Feeding trial 17 | 21 | 19 | 8 | 48 |
| Feeding trial 18 | 11 | 24 | 12 | 47 |
| Feeding trial 19 | 12 | 30 | 6 | 48 |
| Feeding trial 20 | 20 | 21 | 6 | 47 |
| Feeding trial 21 | 31 | 67 | 19 | 117 |
| Feeding trial 22 | 6 | 30 | 12 | 48 |
| Feeding trial 23 | 14 | 29 | 5 | 48 |
| Feeding trial 24 | 12 | 28 | 6 | 46 |
